# Transición en salud mental desde la infancia a adultos: Una revisión exploratoria

**DOI:** 10.23938/ASSN.1107

**Published:** 2025-02-07

**Authors:** Adalia del Carmen Guerra-Ortega, Claudio-Alberto Rodríguez-Suárez, Héctor González-de la Torre

**Affiliations:** 1 Universidad de Las Palmas de Gran Canaria (ULPGC) Facultad de Ciencias de la Salud Departamento de Enfermería Las Palmas de Gran Canaria Canarias España; 2 Complejo Hospitalario Universitario Insular Materno Infantil (CHUIMI) Unidad de Apoyo a la Investigación Las Palmas de Gran Canaria Canarias España

**Keywords:** Psiquiatría Infantil, Psiquiatría del Adolescente, Transición a la Atención de Adultos, Continuidad de Cuidados, Servicios de Salud Mental, Child Psychiatry, Adolescent Psychiatry, Transition to Adult Care, Continuity of Patient Care, Mental Health Services

## Abstract

**Fundamento:**

En adolescentes con trastornos mentales, el control de la enfermedad puede empeorar durante su transición a servicios de adultos. El objetivo del estudio es evaluar el impacto de intervenciones y programas de transición entre servicios de salud mental infanto-juveniles y de adultos en la continuidad del tratamiento y en los resultados de salud mental.

**Metodología:**

Revisión exploratoria mediante búsqueda de los descriptores “Psiquiatría Infantil”, “Psiquiatría del Adolescente”, “Transición a la Atención de Adultos”, “Servicios de Salud Mental” y “Continuidad de la Atención al Paciente” en Medline, *Web of Science*, Scopus, CINAHL, Biblioteca Cochrane, LILACS y CUIDEN realizadas entre octubre y diciembre de 2023. Se incluyeron estudios de investigación con cualquier diseño, en inglés y español, sin límite temporal. La calidad se evaluó con las herramientas del Instituto Joanna Briggs.

**Resultados:**

Se incluyeron ocho estudios (cuatro cualitativos, dos de cohortes y dos revisiones), de calidad moderada a excelente. El 23,5% de los casos lograron una transición exitosa, aunque con retrasos y menor seguimiento a largo plazo, facilitada por transferencia de información, atención paralela y participación de pacientes y familias, y dificultada por alta voluntaria o consumo de drogas previo al ingreso hospitalario.

**Conclusiones:**

Las estrategias clave para optimizar la transición entre servicios de salud mental infanto-juveniles y de adultos incluyen transferencia de información, atención paralela y participación activa. Es esencial implementar medidas coordinadas que aborden barreras, reduzcan retrasos y mejoren el seguimiento, promoviendo procesos anticipados, individualizados y corresponsables entre pacientes, familias y profesionales para promover una atención integral y efectiva.

## Abstract (en)

**Background:**

The management of mental health conditions may deteriorate in adolescents during the transition from adolescent mental health services to adult mental health services. This study aimes to assess the impact of interventions and transition programs on treatment continuity and mental health outcomes for individuals moving between these services.

**Methods:**

A scoping review was conducted using the descriptors “Child Psychiatry”, “Adolescent Psychiatry”, “Transition to Adult Care”, “Mental Health Services”, and “Continuity of Patient Care” in Medline, Web of Science, Scopus, CINAHL, Cochrane Library, LILACS, and CUIDEN, from October to December 2023. Studies of any design published in English and Spanish were included, with no time restrictions. The quality of the studies was assessed using Joanna Briggs Institute tools.

**Results:**

Eight studies were included (two reviews, four qualitative studies, and two cohort studies), with quality ratings ranging from moderate to excellent. Only 23.5% of young people experienced a successful transition, with many facing delays and reduced long-term follow-up. Key factors for a successful transition included the transfer of information, parallel care, and the involvement of patients and family, while voluntary discharge and prior drug use before hospitalization hindered the transition process.

**Conclusions:**

To optimize the continuity of care during the transition from child/adolescent to adult mental health services, key strategies include the transfer of information, parallel care, and active participation. Implementing coordinated measures that address barriers, reduce delays, and enhance follow-up is essential. A proactive, individualized, and collaborative approach involving patients, families, and professionals is necessary to ensure comprehensive and effective care.

## INTRODUCCIÓN

La salud mental es definida, según la Organización Mundial de la Salud (OMS), como un estado de bienestar mental que permite a las personas hacer frente a los momentos de estrés de la vida, desarrollar habilidades, aprender y trabajar adecuadamente y contribuir a la mejora de su comunidad^1^, existiendo determinantes individuales, sociales y ambientales que pueden protegerla o dañarla. Los factores individuales incluyen aspectos psicológicos y biológicos tales como condicionantes genéticos, habilidades emocionales o abuso de sustancias tóxicas. Los riesgos sociales incluyen exposición a la pobreza, violencia y desigualdad, siendo particularmente perjudiciales los factores ocurridos en la primera infancia, tales como el acoso escolar o los castigos físicos^2^^,^^3^. No obstante, estos factores sociales también pueden incluir elementos de protección: interacciones positivas, aspectos educativos, empleo de calidad y vivir en entornos seguros^2^. También existen factores ambientales perjudiciales, como la calidad deficiente del aire, las temperaturas extremas, el ruido y el cambio climático^3^.

En la población infantil, las enfermedades mentales constituyen alteraciones clínicas de la cognición, la regulación de las emociones o el comportamiento^4^. Incluyen diferentes procesos patológicos: *Ansiedad*, con alta prevalencia entre adolescentes, que afecta al rendimiento académico y altera la esfera social y familiar^4^^,^^5^; *depresión*, uno de los principales factores de riesgo de suicidio en adolescentes, y que puede asociarse al abuso de alcohol y/o drogas, a trastornos de la alimentación y al *bulliyng*^4^^,^^5^; *trastorno por déficit de atención e hiperactividad* (TDAH), la alteración del neurodesarrollo más frecuente en la infancia, caracterizado por un patrón persistente de dificultad para prestar atención y/o hiperactividad-impulsividad^4^^,^^6^; *trastorno bipolar*, infrecuente en la infancia pero se estima que los TDAH y otros trastornos de conducta en la infancia son presentaciones iniciales de un futuro trastorno bipolar^7^; *trastornos generalizados del desarrollo* (TGD), determinados por perturbaciones graves de las habilidades de interacción social y comunicación o por la presencia de comportamientos, intereses y actividades estereotipadas; *trastorno del espectro autista* (TEA)^3^^,^^4^; *trastornos disociales*, que incluyen patrones repetitivos y persistentes de comportamientos agresivos o retadores que se inician antes de los diez años debido a factores como maltrato infantil, fracaso académico o exposición a la violencia^3^^,^^4^; *esquizofrenia*, que aparece entre los 16 y 30 años, caracterizada por alucinaciones -las auditivas son más frecuentes en adolescentes que en adultos^4^-, delirios y trastornos del pensamiento; y *trastornos de la conducta alimentaria* (TCA), con conducta alterada ante la ingesta de alimentos o aparición de comportamientos obsesivos sobre el control del peso que afectan significativamente al bienestar emocional, psicosocial y físico de las personas en la adolescencia^8^.

La infancia y adolescencia son etapas que implican cambios físicos, emocionales y sociales favorecedores de la aparición de enfermedades mentales^4^^,^^9^, siendo los 14 años la edad habitual de aparición^2^^,^^9^. Aproximadamente, uno de cada siete adolescentes entre 10 y 19 años padece algún trastorno mental, con una prevalencia mundial del 5-20% de trastorno mental grave y casi un 33% de depresión^2^^,^^4^. Los síntomas tempranos habituales son irritabilidad persistente, ira, aislamiento social y cambios en el apetito o sueño, que en algunas ocasiones no se detectan con antelación, pudiendo repercutir en el rendimiento escolar y las relaciones sociales, con intentos de autolisis en casos extremos. Además, estos adolescentes son vulnerables a sufrir discriminación y estigmatización, lo que retrasa la búsqueda de ayuda especializada y pospone la instauración terapéutica temprana^2^^,^^9^.

Aunque la atención primaria (AP) constituye un punto de partida para el abordaje de los problemas de salud mental, el contexto clínico de la salud mental opera a través de una red de atención especializada, tanto para la atención infanto-juvenil como la de adultos, incluyendo unidades primarias de salud mental, unidades de rehabilitación y estancia intermedia, unidades de internamiento breve (UIB), hospitales infanto-juveniles y centros de día^10^^,^^11^.

En términos generales, la continuidad asistencial requiere establecer una coordinación y comunicación efectivas entre los niveles asistenciales de AP y secundaria (hospitalaria y extrahospitalaria)^12^, que asegure la continuidad de la información, de la gestión y de la relación^13^; la continuidad se pierde cuando un centro desatiende o relega la información sobre pacientes o cuidadores omitiéndola, duplicándola o contradiciéndola^14^^,^^15^.

En el contexto de la salud mental, el proceso de transición se define como el movimiento deliberado y planificado de personas adolescentes y adultas jóvenes con patologías mentales crónicas desde un sistema de atención orientado a la infancia hacia otro orientado a adultos, lo que constituye un proceso complejo y prolongado que requiere atención multidimensional y multidisciplinar^16^. Esta transición exige desarrollar distintos dispositivos que conforman una amplia red sanitaria e implica una importante carga burocrática que coincide con los cambios propios de la adolescencia y conlleva un período de adaptación en el que se experimentan crisis y avances, tanto por parte del adolescente como de su familia. Durante este período se completa el desarrollo de la personalidad y se organizan los patrones de interacción social, surgiendo sentimientos de pérdida de referentes, abandono, injusticia e incomprensión. Así, la transición hacia los servicios de salud mental de adultos constituye un momento crucial para mantener la alianza terapéutica en este entramado debido a la alta vulnerabilidad y riesgo de desestabilización psicopatológica, que pueden reactivar traumas pasados y conducir a la interrupción de los procesos terapéuticos o inicio del consumo de sustancias tóxicas que agravan las patologías mentales^17^. El propósito de la transición es garantizar la continuidad asistencial de los pacientes, permitiéndoles mantener su nivel de salud y máximo potencial como individuos integrados en la sociedad. Las disparidades en la organización de los servicios de salud mental para niños y adolescentes, en comparación con adultos, son claves para conocer si la transición es óptima^15^.

Para abordar la complejidad de la transición en el ámbito clínico de la salud mental en población infanto-juvenil, se ha planteado una revisión de alcance con el objetivo de evaluar las intervenciones y los programas de transición de niños y adolescentes entre servicios de salud mental infanto-juveniles y de adultos, con el fin de examinar su impacto en la continuidad del tratamiento y en los resultados de salud mental.

## MATERIAL Y MÉTODOS

### Diseño

Se realizó una revisión exploratoria ajustada a las normas *Preferred Reporting Items for Systematic Reviews and Meta-Analyses for Scoping Reviews (PRISMA-ScR)*^18^. Para responder a la pregunta de revisión ¿Cuál es la efectividad de los programas de transición entre servicios de salud mental infanto-juveniles y de adultos en niños y adolescentes, en términos de continuidad del tratamiento y mejoras en los resultados de salud mental? Se siguió la propuesta metodológica del Instituto Joanna Briggs (JBI) de plantear la pregunta con formato PIO:

- P (población): población infantil y adolescente en transición desde los servicios de salud mental pediátricos a los de adultos;
- I (intervención): intervenciones o programas diseñados específicamente para facilitar la transición desde los servicios de salud mental pediátricos a los de adultos;
- O (resultados/*outcomes*): continuidad en el tratamiento, mejora de la salud mental con relación a la adherencia al tratamiento, reducción de síntomas y mejora en la calidad de vida.

El protocolo de la revisión no ha sido registrado.

### Estrategia de búsqueda y bases de datos

Inicialmente, se consultó el *International prospective register of systematic reviews (PROSPERO)* para comprobar la existencia de otras revisiones registradas o en proceso de elaboración sobre el tema a estudio. Tras esta consulta inicial, se realizaron búsquedas en las bases de datos *Medline, Web of Science (WoS), Scopus, CINAHL, Biblioteca Cochrane, LILACS* y *CUIDEN*.

Las búsquedas fueron realizadas entre el 31 de octubre y 12 de diciembre de 2023 utilizando los descriptores DeCS (MeSH): “Psiquiatría del Adolescente” (“*Adolescent Psychiatry*”), “Psiquiatría infantil” (“*Child Psychiatry*”), “Continuidad de la atención al paciente” (“*Continuity of Patient Care*”), “Servicios de Salud Mental” (“*Mental Health Services*”) y “Transición a la atención de adultos” (“*Transition to Adult Care*”) y sus términos alternativos, combinados con los operadores booleanos *AND* y *OR*. Las búsquedas fueron realizadas por una investigadora (AdCGO) y verificadas por un segundo investigador (CARS) siguiendo los criterios de la extensión PRISMA-S^19^. La estrategia de búsqueda fue pilotada en *Medline* (a través de *PubMed*) y posteriormente adaptada a las características de cada una de las bases de datos, tal como se muestra en la Tabla 1.

**Tabla 1:** Estrategias de búsqueda en las distintas bases de datos

Base de datos	Estrategia de búsqueda
Fecha de búsqueda	
Medline - 31/10/2023	(((“Child Psychiatry”[Mesh] *OR* “Psychiatry, Child”) *OR* (“Adolescent Psychiatry”[Mesh] *OR* “Psychiatry, Adolescent”)) *AND* ((“Transition to Adult Care”[Mesh] *OR* “Transfer from Pediatric to Adult Care” *OR* “Pediatric Transition To Adult Care” OR “Transferring to Adult Care” *OR* “Transfer to Adult Care”) *OR* (“Continuity of Patient Care”[Mesh] *OR* “Care Continuity, Patient” *OR* “Patient Care Continuity” OR “Continuum of Care” *OR* “Care Continuum” *OR* “Continuity of Care” *OR* “Care Continuity”))) *AND* (“Mental Health Services”[Mesh] *OR* “Health Services, Mental” *OR* “Health Service, Mental” *OR* “Mental Health Service” *OR* “Service, Mental Health” OR “Services, Mental Hygiene” *OR* “Hygiene Service, Mental” *OR* “Hygiene Services, Mental” *OR* “Mental Hygiene Service” *OR* “Service, Mental Hygiene” *OR* “Mental Hygiene Services” OR “Services, Mental Health”)
Scopus 31/10/2023	“Adolescent Psychiatry” *OR* “Child Psychiatry” *AND* “Transition to Adult Care” *OR* “Continuity of Patient Care” *AND* “Mental Health Services”
WoS 02/11/2023	“Adolescent Psychiatry” (All Fields) *OR* “Child Psychiatry” (All Fields) *AND* “Transition to Adult Care” (All Fields) *OR* “Continuity of Patient Care” (All Fields) *AND* “Mental Health Services” (All Fields)
Biblioteca Cochrane 31/10/2023	“Adolescent Psychiatry” (All Fields) *OR* “Child Psychiatry” (All Fields) *AND* “Transition to Adult Care” (All Fields) *OR* “Continuity of Patient Care” (All Fields) *AND* “Mental Health Services”
LILACS 31/10/2023	(“Adolescent Psychiatry”) *OR* (“Child Psychiatry”) *AND* (“Transition to Adult Care”) *OR* (“Continuity of Patient Care”) *AND* (“Mental Health Services”)
CUIDEN 30/10/2023	“Adolescent Psychiatry” *OR* “Child Psychiatry” *AND* “Transition to Adult Care”
CINAHL 12/12/2023	(“Adolescent Psychiatry”) *OR* (“Child Psychiatry”) *AND* (“Transition to Adult Care”) *OR* (“Continuity of Patient Care”) *AND* (“Mental Health Services”)

WoS: *Web of Science*; LILACS: Literatura Latinoamericana y del Caribe en Ciencias de la Salud.

### Criterios de inclusión y exclusión

Se incluyeron estudios con cualquier enfoque metodológico y diseño de investigación (cualitativo, y cuantitativo experimental u observacional) y, dada la perspectiva exploratoria de esta revisión para conocer el estado de la ciencia en el área, revisiones narrativas, exploratorias y sistemáticas, publicados en inglés o español, sin establecer límites para el año de publicación. En cuanto a la población, se seleccionaron estudios que examinaran el proceso de transición acerca de cualquier trastorno mental en población infanto-juvenil, incluyendo las perspectivas de pacientes, familiares o profesionales. Se excluyeron artículos de opinión, editoriales y cartas al editor, además de la literatura gris y los protocolos de estudios de investigación.

### Proceso de cribado

Los registros recuperados fueron exportados a hojas de Excel^®^ para el proceso de selección. Tras eliminar los duplicados, se procedió al cribado por título y resumen. Los que se ajustaron a criterios de inclusión fueron recuperados a texto completo.

La calidad de los registros seleccionados se evaluó utilizando las herramientas de lectura crítica de JBI apropiadas a cada uno de los diseños^20^. De acuerdo a su tasa de cumplimiento, los estudios se categorizaron en calidad baja (≤69%), moderada (70-79%), alta (80-89%) y excelente (≥90%). Para ser incluidos en el proceso de revisión, cada estudio debía alcanzar una tasa de cumplimiento igual o superior al 70% de ítems con respuesta afirmativa en las herramientas JBI. Para los estudios observacionales (ocho ítems) se precisaron al menos seis respuestas afirmativas, para los estudios cualitativos (nueve ítems) al menos seis respuestas afirmativas y para los estudios de revisión (once ítems) al menos ocho ítems con respuesta afirmativa. El cribado se realizó mediante revisión por pares (AdCGO y CARS) y, en caso de discrepancias, decidió el tercer investigador (HGdlT). Se realizó un pilotaje con una muestra de registros para verificar la idoneidad del proceso.

### Proceso de extracción de datos y síntesis de resultados

Se extrajeron las variables bibliométricas y sociodemográficas de los estudios incluidos, así como la información estadística correspondiente a las variables clínicas relacionadas con la continuidad asistencial de niños y adolescentes en el ámbito clínico de la salud mental. Además, se obtuvo la siguiente información de los estudios: país y año de publicación, diseño del estudio, detalles sobre el tamaño y características de la población, características de los instrumentos utilizados para evaluar la continuidad asistencial y resultados principales y secundarios alcanzados. Para las variables cuantitativas se recopilaron medias (M), desviaciones estándar (DE) e intervalos de confianza (IC95%) y, para las cualitativas, frecuencias (n) y porcentajes (%); también se extrajo el valor de significación de los contrastes de hipótesis (p) y los tamaños de efecto (TE) cuando estaban disponibles. De los estudios cualitativos se extrajeron los temas y subtemas identificados. La extracción de datos fue realizada de forma independiente por dos investigadores (AdCGO y CARS) y el tercer investigador (HGdlT) resolvió todas y cada una de las discrepancias. Para la extracción de datos se utilizó el gestor de referencias Mendeley^®^, realizándose un pilotaje a una muestra de los estudios.

## RESULTADOS

**Figura 1:**
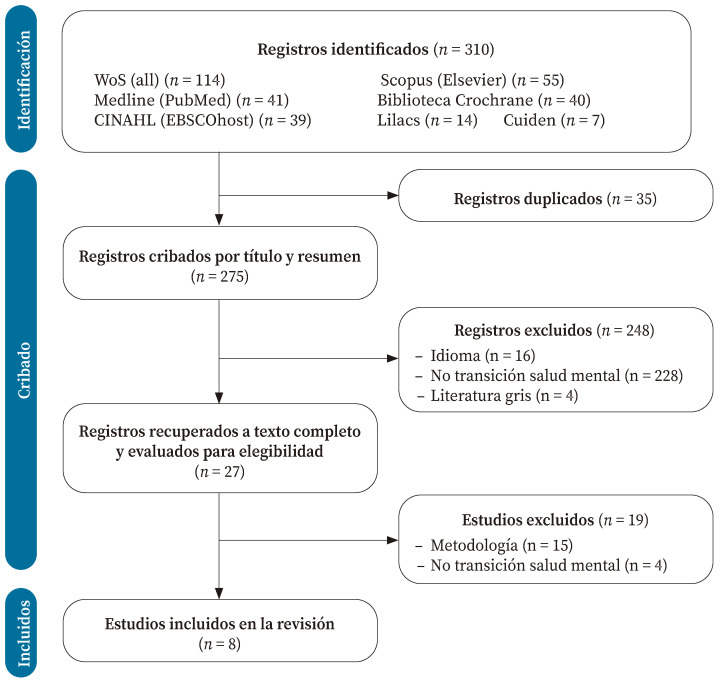
Diagrama de flujo de selección de los estudios incluidos*.

Se identificaron 310 registros. Tras eliminar los duplicados, los restantes se cribaron mediante lectura de título y resumen. Los 27 registros restantes se revisaron a texto completo, eligiendo ocho estudios para ser incluidos en la revisión^21^^-^^28^ (Fig. 1).

* Page y col. BMJ 2021; 372: n71. https://doi.org/10.1136/bmj.n71

Los estudios incluidos eran dos revisiones (una de alcance^25^ y una sistemática^27^), cuatro estudios cualitativos^22^^,^^24^^,^^26^^,^^28^ y dos observacionales de cohortes^21^^,^^23^. Canadá^22^^,^^25^^,^^26^ y Reino Unido^24^^,^^27^^,^^28^ contribuyeron con tres estudios cada uno. Hubo un estudio observacional multicéntrico^21^.

Todos los estudios obtuvieron puntuaciones JBI entre el 72,7 y el 81,8%, por lo que los ocho fueron incluidos en la revisión. La calidad de la revisión de alcance^25^ fue alta, mientras que la del resto de estudios^21^^,^^22^^-^^28^ fue moderada. Los estudios de Appleton y col^21^^,^^24^^,^^27^, Pontoni y col^23^ y Cleverly y col^25^ destacaron por la claridad en la definición de los criterios de inclusión, mientras que las debilidades estuvieron en el control de sesgos y de factores de confusión. Los estudios de Ghanouni y col^22^, Appleton y col^24^, Cleverly y col^25^ y Belling y col^28^ mostraron fortalezas en la congruencia metodológica y adecuada representación de los participantes, aunque algunas áreas de mejora incluyeron la influencia del investigador y el contexto cultural (Tabla 2).

**Tabla 2:** Calidad de los estudios incluidos en la revisión valorada mediante herramientas de lectura crítica del Instituto Joanna Briggs^20^

Autor	P1	P2	P3	P4	P5	P6	P7	P8	P9	P10	P11	Score
Año												(%)
*Revisiones*												*11*
Cleverley y col	S	S	S	S	S	S	S	N/A	N/A	S	S	9
2020^25^												(81,8)
Appleton y col	S	S	S	S	S	S	N	N/A	N/A	S	S	8
2019^27^												(72,7)
*Cualitativos*												*7*
Ghanouni y col	S	S	S	S	N	N	S	S	S	-	-	7
2022^22^												(77,7)
Appleton y col	S	S	S	S	N	N	S	S	S	-	-	7
2021^24^												(77,7)
Cleverley y col	S	S	S	S	N	N	S	S	S	-	-	7
2020^26^												(77,7)
Belling y col	S	S	S	S	N	S	S	N	S	-	-	7
2014^28^												(77,8)
*Cohortes*												*8*
Appleton y col	S	S	S	S	N	N	S	S	-	-	-	6
2023^21^												(75)
Pontoni y col	S	S	S	S	N	N	S	S	-	-	-	6
2022^23^												(75)

S: sí; N: no; N/A: no aplica.

*Revisión:* **P1** ¿La pregunta de la revisión es clara y explícita?; **P2** ¿Los criterios de inclusión son apropiados para la pregunta de revisión?; **P3** ¿Fue apropiada la estrategia de búsqueda?; **P4** ¿Fueron adecuadas las fuentes y los recursos utilizados para la búsqueda de estudios?; **P5** ¿Fueron adecuados los criterios de valoración de los estudios?; **P6** ¿La valoración crítica fue realizada por dos o más revisores de forma independiente?; **P7** ¿Hubo métodos para minimizar los errores en la extracción de datos?; **P8** ¿Fueron adecuados los métodos utilizados para combinar los estudios?; **P9** ¿Se evaluó la probabilidad de sesgo de publicación?; **P10** ¿Las recomendaciones para la política y/o la práctica estaban respaldadas por los datos comunicados?; **P11** ¿Las directivas específicas para la nueva investigación fueron apropiadas?

*Cualitativo:* **P1** ¿Existe congruencia entre la metodología de la investigación y la pregunta u objetivos de la misma?; **P2** ¿Existe congruencia entre la metodología de la investigación y los métodos utilizados para recoger los datos?; **P3** ¿Existe congruencia entre la metodología de investigación y la representación y el análisis de los datos?; **P4** ¿Existe congruencia entre la metodología de la investigación y la interpretación de los resultados?; **P5** ¿Hay alguna afirmación que sitúe al investigador en el plano cultural o teórico?; **P6** ¿Se aborda la influencia del investigador en la investigación, y viceversa?; **P7** ¿Están los participantes, y sus expresiones, adecuadamente representados?; **P8** ¿Cumple la investigación con los criterios éticos o hay pruebas de aprobación ética por un organismo adecuado?; **P9** ¿Las conclusiones del informe de investigación se derivan del análisis o la interpretación de los datos?

*Cohortes:* **P1** ¿se definieron claramente los criterios de inclusión en la muestra?; **P2** ¿Se describieron detalladamente los sujetos del estudio y el entorno?; **P3** ¿Se midió la exposición de forma válida y fiable?; **P4** ¿Se utilizaron criterios objetivos y normalizados para medir la condición?; **P5** ¿Se identificaron los factores de confusión?; **P6** ¿Se indicaron las estrategias para hacer frente a los factores de confusión?; **P7** ¿Se midieron los resultados de forma válida y fiable?; **P8** ¿Se utilizó un análisis estadístico adecuado?

Las revisiones incluidas abordaron la complejidad de la transición entre los servicios de atención pediátrica y adulta, analizaron diferentes efectos en la atención sanitaria, los elementos clave para lograr una transición exitosa y los resultados de su implementación. La revisión de alcance^25^ incluyó 86 estudios e identificó cuatro criterios esenciales para una transición óptima y seis elementos fundamentales que deberían estar presentes en todo proceso de transición, detectando que la participación activa de jóvenes y sus familias en la toma de decisiones favorece el éxito de la transición. Con un enfoque complementario, la revisión sistemática^27^ realizada sobre 13 estudios de diseño heterogéneo identificó los motivos que dificultaban la transición hacia los servicios de salud mental para adultos, ya que ni una cuarta parte de los jóvenes lograron realizar una transición exitosa, con un retraso en la atención y una disminución de la tasa de seguimiento entre el primer y tercer año post-transición^27^ (Tabla 3).

**Tabla 3:** Características de los estudios de revisión

Autor / Año / País	Diseño / Objetivo / Artículos / Características	Resultados
Cleverley y col^25^	- Revisión de alcance	- 4 criterios para una transición óptima: transferencia de información, período de atención paralela, planificación de la transición, continuidad de cuidados
2020	- Identificar intervenciones que apoyen el éxito de la transición	- 6 elementos básicos de transición: política, seguimiento y control, preparación y planificación de la transición, transferencia de la asistencia y finalización de la transferencia
Canadá	- n=86	- 1 elemento especial de transición: participación de jóvenes/familias en la toma de decisiones
	• n=56 revisados por pares	*Conclusión:* La coordinación integrada, con participación activa de jóvenes y familias, mejora la transición
	• n=30 literatura gris	
	- Temática:	
	• salud mental general (n=67)	
	• TDAH (n=15)	
	• trastornos alimentarios (n=4)	
	- No especifica sexo ni n de pacientes	
Appleton y col^27^	- Revisión sistemática	- Transición exitosa: 1.255 jóvenes (23,46%)
2019	- Sintetizar y revisar la investigación sobre la transición	- Seguimiento por adultos: 115 (86%) con 1 cita; 16% alta tras 1 cita; 55% alta entre 1 y 3 años posteriores
Reino Unido	- n=13	- Alta en adultos (n=103)
	• n=10 cohortes	- Retraso en tiempo de espera (55-110 días)
	• n=3 transversales	- No remitidos/alta (n=12)
	- n=5.350 jóvenes, no especifica sexo	- Desvinculados (n=92)
	- Países: Canadá, Reino Unido, Irlanda, Francia, Australia.	- Pendientes de transición (n=46)

TDAH: Trastorno por déficit de atención e hiperactividad.

Los estudios cualitativos identificaron diferentes temas y subtemas relacionados con las vivencias y experiencias a partir de entrevistas en profundidad de duración entre 30 y 60 minutos a una muestra de entre 20 y 25 participantes, que incluyeron progenitores^22^^,^^24^, jóvenes^24^^,^^26^ o profesionales^22^^,^^28^ de diferentes categorías, en los ámbitos de hospitalización^22^^,^^24^^,^^26^ y centros comunitarios^22^. Dos estudios^26^^,^^28^ no indicaron el sexo de los participantes. Los hallazgos incluyeron temas coincidentes relativos a la calidad de la atención^22^^,^^24^, preocupaciones por la brecha asistencial^24^^,^^26^ y barreras o conflictos durante el proceso de transición^22^^,^^24^^,^^26^^,^^28^. Los estudios concluyeron, de manera similar, la necesidad de disminuir las barreras burocráticas^22^ y abordar nuevos modelos de atención^24^, así como diseñar directrices flexibles^28^ y adaptadas a las perspectivas de los pacientes^26^. En la Tabla 4 se muestran las características de los participantes, el contexto clínico y la técnica de recogida de datos, así como los temas y subtemas identificados.

**Tabla 4:** Características de los estudios de diseño cualitativo

Autor	Objetivo	Temas
Año	Participantes (n y sexo)	Subtemas
País	Contexto clínico	Conclusiones
	Técnica de recogida de datos	
Ghanouni y col ^22^	- Investigar las experiencias y perspectivas sobre barreras en jóvenes con TEA	*Temas:*
2022	- n=20	- Accesibilidad y calidad de la atención
Canadá	• n=17 progenitores (16 mujeres)	- Tensiones y conflictos
	• n=3 profesionales (2 mujeres)	- Navegación y atención integrada
	- Centros comunitarios, clínicas y organizaciones de autismo	*Subtemas:*
	- Entrevistas semiestructuradas de 60 minutos	- Atención especializada: coste, lista de espera, ubicación geográfica
		- Cambio de tutela, clasificación de funcionamiento, elegibilidad de servicios
		- Acceso a información y planificación
		*Conclusiones:*
		- La falta de servicios especializados y barreras burocráticas dificultan la atención
Appleton y col ^24^	- Investigar las causas de la pérdida de continuidad	*Temas:*
2021	- n=25 entrevistas en profundidad (10 mujeres)	- Barreras sistémicas para la continuidad
Reino Unido	• n=20 individuales	- Problemas con la calidad de la atención
	• n=5 conjuntas progenitor/hijo	- Efectos de caer en la brecha asistencial
	- Hospitalización: servicios de salud mental	*Subtemas:*
	- Entrevistas en profundidad individuales de 36 minutos y conjuntas de 56 minutos	- No estar suficientemente enfermo
		- Prestación de servicios inadecuada
		- Falta de atención conjunta
		- No recibir atención adecuada
		- No estar preparado para finalizar en los servicios infantiles
		- Posibilidad de acceder a más atención
		- Efectos en los jóvenes: sentirse abandonado, arreglárselas sin atención, problemas con la medicación
		- Efectos en los progenitores: impacto emocional, asumir un papel activo
		*Conclusiones:*
		- La falta de continuidad provoca ansiedad. Los progenitores sin apoyo asumen el cuidado. Es preciso abordar nuevos modelos de atención
Cleverley y col ^26^	- Explorar los conocimientos, expectativas y vivencias de los jóvenes.	*Temas:*
2020	- n=21 jóvenes	- Cambio de significado de la “transición”
Canadá	• n=20 en seguimiento por servicios de adultos	- Preparación para la transición
	• n=1 no en seguimiento	- Reacciones ante la edad de transición
	• n=13 pre-transición	- Falta de información, preparación e implicación
	• n=8 post-transición	- Confusión de roles y responsabilidades
	- Hospitalización: servicios de salud mental infantil	- Preocupación por la brecha asistencial
	- Entrevistas semiestructuradas de 30-60 minutos	- Recomendaciones para sistema y profesionales
		- Recomendaciones para jóvenes y familias
		*Subtemas:*
		- Asociarse con los jóvenes en la planificación
		- Propósito de la transición
		- Garantizar el acceso a la información
		- Garantizar la continuidad entre servicios
		- Recomendaciones adicionales
		*Conclusiones:*
		- Es preciso codiseñar directrices de transición adaptadas, integrando la perspectiva juvenil
Belling y col ^28^	- Investigar factores organizacionales para la transición.	*Temas:*
2014	- n=34 profesionales	- Cuestiones de elegibilidad
Reino Unido	• (8 enfermeras, 6 psiquiatras, 2 psicólogos, 7 trabajadores sociales, 7 gerentes, 4 otros).	- Recursos
	- Sistema Nacional de Salud (n=30 centros: 16 infantiles; 11 de adultos; 3 mixtos, 4 voluntariado)	*Subtemas:*
	- Entrevistas semiestructuradas telefónicas de 40-60 minutos	- Falta de claridad sobre la disponibilidad del servicio y los criterios de elegibilidad
		- Diferencias entre servicios
		- Los servicios para adultos no aceptan pacientes hasta 17 o 18 años, variabilidad en edades
		- Cargas de trabajo en los servicios para adultos
		- Los servicios no satisfacen necesidades más allá de las enfermedades mentales graves, problemas de aprendizaje y TDAH/TEA
		*Conclusiones:*
		- La falta de coordinación y de criterios estrictos interrumpe la transición; se necesitan criterios flexibles y servicios adaptados

TEA: trastorno de espectro autista; TDAH: trastorno por déficit de atención e hiperactividad

Los estudios observacionales de cohortes identificaron y analizaron factores predictores de adecuada e inadecuada transición desde salud mental infanto-juvenil a adultos^21^^,^^23^, así como factores predictores de mayor y menor coste económico^21^. En el estudio retrospectivo de Pontoni y col^23^, se recogieron datos de las evaluaciones a pacientes en la última hospitalización infanto-juvenil y a los seis, doce y veinticuatro meses tras su traslado a adultos. El estudio prospectivo de Appleton y col^21^ aplicó distintos instrumentos de evaluación: *Clinical Global Impression Scale* (CGI-S) para la gravedad de la enfermedad (1 ítem; 0=no enfermo a 7=máxima enfermedad), *Nation Outcome Scale for Children and Adolescents* (HoNOSCA) para las necesidades clínicas (15 ítems; 0=no problema a 4=problema muy grave), *Achenbach System of Empirically Based Assessment* (ASEBA) para el comportamiento y problemas emocionales (0=nunca ocurre a 2=ocurre con frecuencia), *Independent Behaviour During Consultation Scale* (IBDCS) para la autoeficacia e independencia (0=nada en absoluto a 3=totalmente independiente) y *Euro Quality of life* (EQ5D-5L) para la calidad de vida relacionada con la salud (0=no tiene problemas a 5=problemas extremos). Para Appleton et al^21^ y Pontoni et al^23^, los factores que mejoraron la transición incluyeron menor severidad de la enfermedad, incremento de las necesidades clínicas, mayor autoeficacia e independencia de los pacientes, evento de primera hospitalización con edad mayor de 16 años, ingresos hospitalarios superiores a 41 días, terapia con fármacos, prescripción de antipsicóticos atípicos y diagnóstico de esquizofrenia u otros trastornos psicóticos. Los factores que se asocian a una transición fallida fueron alta voluntaria y consumo de drogas antes de la hospitalización (Tabla 5).

**Tabla 5:** Características de los estudios observacionales

Autor	Diseño	Variable de resultado
Año	Objetivo	Factores predictores (OR; IC95%; p)
País	Muestra	Conclusiones
	Características	
	Instrumentos	
Appleton y col^21^	- Cohortes prospectivo	- Transición exitosa desde la salud mental infantil a adultos: n=152 (31,1%), predominio femenino (n=98; 64,47%), sin diferencias de edad
2023	- Identificar factores que dificultan la transición y analizar costes	- *Adecuada transición*. se asociaron significativamente: mayor severidad de la enfermedad (CGI-S) (4,32; 1,19-15,65; p=0,017), mayores necesidades clínicas (HoNOSCA) (1,06; 1,02-1,10; p=0,002), y mayor autoeficacia e independencia (IBDCS) (1,05; 1,01-1,09; p=0,03)
Reino Unido, Bélgica, Croacia, Francia, Alemania, Irlanda, Italia, Países Bajos	- n=488	- *Transición inadecuada*. Se asociaron de forma no significativa: sexo masculino, mayor tiempo de seguimiento infantil e intentos de suicidio
	• 58,2% mujeres	- *Coste económico*: transición adecuada e intentos de suicidio se asociaron (p < 0,01) a mayor coste; mejor CdV, T. emocional y seguimiento infantil prolongado se asociaron (p ≤ 0,01) a menor coste
	• edad M=17 años	*Conclusiones*: solo jóvenes con enfermedad mental grave completan la transición. Los costes bajan tras la transición
	- Instrumentos:	
	• CGI-S,	
	• HoNOSCA,	
	• ASEBA,	
	• IBDCS,	
	• EQ5D-5L	
Pontoni y col^23^	- Cohortes retrospectivo con registros en dos etapas: última hospitalización infantil (T0/basal) y tras la transición (T1/ 6 meses, T2/ 12 meses; T3/ 24 meses)	- Factores asociados al traslado exitoso a los servicios de salud mental para adultos
2022	- Examinar la transición de niños y adolescentes hospitalizados	- *Adecuada transición*. se asociaron significativamente: edad >16 años en T0 (3,70; 1,86-8,60; p<0,01), estancia hospitalaria >41 días (2,2; 1,1-4,4; p=0,02), monoterapia (4,0; 1,0-16,0; p=0,04) o politerapia con fármacos (10,2; 2,7-38,0; p<0,01), antipsicóticos atípicos (4,0; 1,9-8,6; p <0,01), esquizofrenia y T. psicóticos (4,0; 1,2-8,3; p <0,01)
Italia	- n=322	- *Transición inadecuada*. alta voluntaria (0,2; 0,1-1,3; p=0,09). Se asociaron de forma no significativa: divorcio de progenitores, antecedentes familiares psiquiátricos, múltiples ingresos, cambio de institución sanitaria, alta voluntaria, prescripción de benzodiacepinas al alta, consumo de drogas antes de la hospitalización, T. neurótico, apoyo escolar del profesor y absentismo escolar
	• M=15,1 años (DE=1,8)	*Conclusiones*: Hospitalización temprana, tratamientos y antecedentes familiares dificultan la transición, aunque este impacto disminuye con el tiempo; se necesitan más estudios sobre factores a largo plazo
	• n=151 (47%) con ingresos previos	
	- Diagnósticos:	
	• T. emocionales (n=77; 24%)	
	• T. neuróticos (n=63; 20%)	
	• esquizofrenia y otros T. psicóticos (n=56; 17%)	
	- Prescripción de fármacos al alta:	
	• n=288 (89%)	
	- Fármacos	
	• antipsicóticos (91,5%)	
	• estabilizadores del humor (28%)	
	• benzodiacepinas (25%)	
	• antidepresivos (18,5%)	

M: media; DE: desviación estándar; OR: *odds ratio*; IC: intervalo de confianza; CGI-S: *Clinical Global Impression Scale*; HoNOSCA: *Nation Outcome Scale for Children and Adolescents*; ASEBA: *Achenbach System of Empirically Based Assessment*; IBDCS: *Independent Behaviour During Consultation Scale*; EQ5D-5L: *Euro Quality of life*; CdV: calidad de vida; T.: trastorno.

## DISCUSIÓN

El proceso de transición de personas jóvenes con trastornos mentales ocurre en un momento crucial de sus vidas al coincidir con múltiples cambios físicos, psicológicos, emocionales, afectivos y sociales, por lo que realizar una adecuada transición es trascendental^29^. Sin embargo, los estudios que abordan esta transición son escasos.

La revisión sistemática de Appleton y col^27^ abordó temas similares a la presente, pero mientras su objetivo se centró en analizar los resultados post-transición, en la presente revisión se adopta una perspectiva más amplia, enfocándose en identificar intervenciones o programas específicos que faciliten la transición, y en examinar el impacto de la transición con relación a la adherencia terapéutica, reducción de síntomas y Calidad de vida. Además, el estudio de Appleton y col^27^ incluyó solamente estudios publicados con anterioridad a 2017, lo que justifica la necesidad de actualizar las evidencias disponibles.

Las dos revisiones incluidas concluyen que los sistemas sanitarios estructurados, que involucran a los pacientes y sus familias en el proceso de transición, mejoran la fluidez y sostenibilidad del proceso^25^^,^^27^. En 2023, la revisión de Markoulakis y col^30^ identificó cinco áreas temáticas con facilitadores para la transición: disponer de apoyos holísticos, preparación proactiva, empoderamiento de jóvenes y familias, relaciones de colaboración y consideraciones sistémicas. Estas áreas implican la adecuación de los servicios de salud a las necesidades específicas de la población infanto-juvenil y la búsqueda de apoyos por parte de pacientes y sus familias, a fin de satisfacer las necesidades de jóvenes y familias proporcionando información contextualizada y mejorando la comprensión de cómo los programas de salud mental pueden apoyar sus necesidades, involucrar a los familiares, reducir las barreras y aprovechar los facilitadores existentes.

Las aproximaciones cualitativas al objeto de estudio profundizan en las experiencias relacionadas con el proceso de transición, la discontinuidad en la atención y los factores organizacionales que afectan una transición adecuada^21^^,^^23^, así como aquellos aspectos que inciden en la desvinculación de la red de salud mental^21^. Además, se observa una variabilidad cultural en relación con la conceptualización del cuidado^30^^-^^32^ y los procesos de continuidad asistencial. Los conceptos y medidas clave relacionados con estos aspectos pueden ser fundamentales para lograr una transición exitosa, como señalan Cleverley y col^25^, quiénes destacan la importancia de mantener una perspectiva amplia al implementar intervenciones que favorezcan el éxito de la transición.

Frente al proceso de transición planificada y progresiva desde las unidades infantiles a adultos siguiendo programas de acompañamiento, coexisten situaciones de transferencias o traspasos directos sin mediar los pasos recomendados por estas guías^33^. En este sentido, las intervenciones para mejorar la transición se han centrado en un número limitado de procesos diagnósticos, sobre todo en diabetes de tipo 1, mientras que otras enfermedades pediátricas comunes, como el asma, no han sido estudiadas^34^. Según Coca y col^33^, las personas con fibrosis quística derivadas desde una unidad pediátrica siguiendo un proceso planificado de transición a una unidad de adultos conocían mejor sus medicamentos, mientras que los pacientes transferidos sin planificación gestionaban mejor sus citas y la toma de decisiones; sin embargo, las personas transferidas poseían peor estado emocional y se sentían más tristes, lo cual es un elemento clave para la adherencia al tratamiento. Además, los sistemas sanitarios han puesto un mayor énfasis en la preparación para la transición hacia los servicios de adultos, prestando menos atención a las necesidades y resultados posteriores al traslado^34^.

Bailey y col^35^ han estudiado la relación entre los indicadores de calidad y los efectos adversos en la salud durante la transición de población infanto-juvenil con distintas enfermedades crónicas y observaron que, aunque la mayoría de los indicadores de calidad estuvieron centrados en el paciente, pocos resultados fueron informados por los propios pacientes o sus progenitores, y ninguno se centró en la equidad, mostrando la necesidad de involucrar más a los pacientes en el proceso de transición^35^. Appleton y col^27^ mostraron que la vinculación y seguimiento por los servicios de adultos fue baja, con transición exitosa en una minoría de jóvenes, señalando la importancia de un correcto seguimiento para evitar la desvinculación de la red de salud mental^25^^,^^27^. Es preciso, por tanto, disponer de mecanismos de seguimiento efectivos para disminuir los tiempos de espera y mantener las tasas de seguimiento durante el período crítico posterior a la transición^27^. Los procesos de seguimiento inadecuados y la ausencia de intervenciones de soporte personalizadas son barreras identificadas por Markoulakis y col^30^. Aunque las políticas y una planificación estructurada son esenciales^25^, también es crucial considerar los aspectos prácticos y las barreras operativas^27^. La combinación de una política estructurada y el seguimiento activo del paciente pueden ser clave para mejorar los resultados, asegurando que las intervenciones respondan a las necesidades específicas de los jóvenes con enfermedades complejas^36^.

Los estudios cualitativos destacan la importancia de la planificación anticipada^22^^,^^26^, del ajuste del límite de la edad de transición^26^^,^^28^, y de la necesidad de definir los criterios de elegibilidad durante la transición^22^^,^^28^. Ghanouni y col^22^ se enfocaron en la transición de jóvenes con TDAH, por ser un sector de población con dificultades para mantener el vínculo asistencial y con barreras sistemáticas que favorecen la brecha asistencial^24^. Asimismo, se destaca la necesidad de aumentar la especialización de los servicios y sus profesionales respecto a estas personas jóvenes con problemas de aprendizaje, TDAH y TEA^22^^,^^28^. Cleverley y col^26^ destacan que está aconteciendo un cambio de conceptualización sobre la *transición* y sus circunstancias influyentes. La meta-síntesis realizada por Ludvigsen y col^37^ muestra hallazgos relevantes sobre las experiencias de los progenitores, señalando sometimiento a presiones cruzadas simbolizadas a través de cuatro temas centrales: fluctuación entre las funciones parentales, navegación por contextos sanitarios contrastados, toma de decisiones ante conflictos internos y confianza en la capacidad de autogestión de sus hijos. Estos autores concluyeron que la transición es una experiencia personal en la que pueden producirse, simultáneamente, múltiples transiciones que implican demandas concurrentes, conflictivas y abrumadoras. Desde el sistema de salud, los profesionales deben ser capaces de reconocer las vivencias y angustias de los progenitores para promover que los traslados sean seguros y predecibles, a través de un proceso de toma de decisiones colaborativo entre las partes implicadas: progenitores, hijos y profesionales^37^.

Disponer de un sistema sanitario con transición estructurada es un factor primordial para mejorar la adherencia, el control específico de la enfermedad, la Calidad de vida, las habilidades de autocuidado, la satisfacción con la atención recibida y el uso adecuado de los recursos sanitarios^38^. Sin embargo, Kallio y col^39^ señalan baja correlación (r <0,3) entre la experiencia de transición y los cambios en la Calidad de vida, si bien, los subdominios social y educativo mejoraron tras la transición. Por tanto, es preciso evaluar con mayor exactitud a los proveedores de cuidados, así como los costes derivados de estos cuidados^38^.

Las limitaciones de la investigación derivan, en primera instancia, de las escasas evidencias disponibles sobre el tema abordado, siendo preciso aumentar el número y rigor de estudios en este ámbito clínico. Esto es, en parte, debido a que muchos de los estudios recuperados inicialmente no abordaban la continuidad asistencial en el contexto específico de la salud mental o población infantil. Por otro lado, es importante mencionar la posibilidad de un sesgo contextual, dado que la mayoría de los estudios proceden del Reino Unido y Canadá, siendo preciso conocer la naturaleza de estos procesos en otros contextos clínicos adicionales.

Por último, es importante resaltar la complejidad inherente a la realización de una revisión exploratoria, la cual abarca diseños heterogéneos. Esto conlleva la necesidad de extraer y analizar resultados de diversa naturaleza, enfrentándose en algunos estudios a información incompleta o a la ausencia de datos relevantes. La variabilidad en la calidad, identificada mediante la lectura crítica, evidenció un nivel metodológico aceptable, con fortalezas en la congruencia metodológica y la validez de las mediciones. No obstante, la persistencia de áreas de mejora debe ser considerada para interpretar adecuadamente los hallazgos y orientar futuras investigaciones.

Las fortalezas de esta revisión ponen de relieve y visibilizan las dificultades derivadas de la división del sistema sanitario en dos niveles asistenciales^10^, especialmente cuando se trata de población vulnerable como niños y jóvenes con enfermedades mentales. El factor predictivo más significativo que afecta al fracaso de la continuidad asistencial son los antecedentes de hospitalización^40^, ya que implican movimientos recurrentes entre los dos niveles asistenciales^10^. Por ello, para mejorar la continuidad asistencial es imprescindible dirigirse hacia un modelo integrado favorecedor de la comunicación entre estos niveles, a fin de reducir el riesgo de desvinculación de la red de salud mental^29^. Para abordar las importantes barreras presentes en los sistemas de salud, educativos y sociales y mejorar la transición hacia adultos en jóvenes con enfermedades crónicas^40^, las recomendaciones disponibles en la literatura incluyen mejorar la continuidad de los cuidados^10^^,^^29^^,^^41^, facilitar la atención centrada en el paciente^41^, crear redes de apoyo sólidas y educar para preparar a las personas jóvenes en los futuros procesos de transición^29^^,^^41^. También es preciso investigar más en el desarrollo de intervenciones de atención centrada en las familias, con mayor implicación de pacientes, y mejorar la información transparente sobre el desarrollo y la implementación de tales intervenciones, especialmente respecto a la aclaración de los objetivos y los procesos de compromiso con pacientes, cuidadores y proveedores de salud^42^. Estas mejoras deben contribuir al proceso de transición hacia la edad adulta en los diferentes ámbitos clínicos^43^ y contextos socioculturales^44^^-^^47^ de personas adultas con enfermedades crónicas iniciadas durante la infancia. Tales intervenciones promueven el apoyo sistemático durante el proceso de transición, fomentan la autonomía del paciente y mejoran su participación social, redundando en el desarrollo de su autonomía e integración social^43^^,^^44^.

En conclusión, la evidencia actual destaca la importancia de optimizar la continuidad asistencial para jóvenes con trastornos mentales durante su transición a los servicios de salud mental para adultos. Entre las estrategias recomendadas se incluyen la transferencia efectiva de información, períodos de atención paralela y la participación activa de los jóvenes y sus familias en la toma de decisiones. Sin embargo, es fundamental aumentar la investigación en este ámbito, e implementar estrategias más sólidas y coordinadas que aborden las barreras en el proceso, reduzcan los retrasos y mejoren el seguimiento a largo plazo. Estas medidas deben orientarse hacia una mayor coordinación, anticipación e individualización de los procesos de transición, permitiendo minimizar complicaciones y garantizar un mejor control y manejo de las patologías. Asimismo, se debe fomentar la corresponsabilidad entre jóvenes, familias y profesionales para promover una atención integral y efectiva.

## Data Availability

Se encuentran disponibles bajo petición al autor de correspondencia.
